# International Alliance of Urolithiasis (IAU) consensus on miniaturized percutaneous nephrolithotomy

**DOI:** 10.1186/s40779-024-00562-3

**Published:** 2024-10-28

**Authors:** Guo-Hua Zeng, Wen Zhong, Giorgio Mazzon, Wei Zhu, Sven Lahme, Sanjay Khadgi, Janak Desai, Madhu Agrawal, David Schulsinger, Mantu Gupta, Emanuele Montanari, Juan Manuel Lopez Martinez, Shabir Almousawi, Vincent Emanuel F. Malonzo, Seshadri Sriprasad, Chu Ann Chai, Vimoshan Arumuham, Stefania Ferretti, Wissam Kamal, Ke-Wei Xu, Fan Cheng, Xiao-Feng Gao, Ji-Wen Cheng, Bhaskar Somani, Mordechai Duvdevani, Kah Ann Git, Christian Seitz, Norberto Bernardo, Tarek Ahmed Amin Ibrahim, Albert Aquino, Takahiro Yasui, Cristian Fiori, Thomas Knoll, Athanasios Papatsoris, Nariman Gadzhiev, Ulanbek Zhanbyrbekuly, Oriol Angerri, Hugo Lopez Ramos, Iliya Saltirov, Mohamad Moussa, Guido Giusti, Fabio Vicentini, Edgar Beltran Suarez, Margaret Pearle, Glenn M. Preminger, Qing-Hui Wu, Otas Durutovic, Khurshid Ghani, Marcus Maroccolo, Marianne Brehmer, Palle J. Osther, Marek Zawadzki, Azimdjon Tursunkulov, Monolov Nurbek Kytaibekovich, Abdusamad Abdukakhorovich Abuvohidov, Cesar Antonio Recalde Lara, Zamari Noori, Stefano Paolo Zanetti, Sunil Shrestha, Jean de la Rosette, John Denstedt, Zhang-Qun Ye, Kemal Sarica, Simon Choong

**Affiliations:** 1 Department of Urology and Guangdong Key Laboratory of Urology, First Affiliated Hospital of Guangzhou Medical University, Guangzhou, 510230 China; 2 Department of Urology, San Bassiano Hospital, 36061 Vicenza, Italy; 3 Department of Urology, Siloah St. Trudpert Hospital, 75179 Pforzheim, Germany; 4 Department of Urology, Vayodha Hospital, Kathmandu, 44600 Nepal; 5 Department of Urology, Muljibhai Patel Urological Hospital, Nadiad, 387001 India; 6 Department of Urology, Centre for Minimally-Invasive Endourology, Global Rainbow Healthcare, Agra, 282007 India; 7 Department of Urology, Stony Brook University School of Medicine, Stony Brook, NY 11794 USA; 8 Department of Urology, Icahn School of Medicine at Mount Sinai, Mount Sinai Health System, New York, NY 10029 USA; 9 Department of Urology, Fondazione IRCSS Ca’ Granda, Ospedale Maggiore Policlinico, University of Milan, 20122 Milan, Italy; 10 Department of Urology, Barcelona Clinical Hospital, 08036 Barcelona, Spain; 11 Department of Urology, Sabah Al Ahmad Urology Centre, 20005 Kuwait, Kuwait; 12 Department of Surgery, Section of Urology, Veterans Memorial Medical Center, 1110 Quezon City, Metro Manila, Philippines; 13 Department of Urology, Darent Valley Hospital, Dartford, DA2 8DA UK; 14 Department of Urology, University of Belgrade, 11120 Belgrade, Serbia; 15 Department of Urology, Stone and Endourology Unit, University College London Hospitals NHS Foundation Trust, London, NW1 2BU UK; 16 Department of Urology, Hospital, University of Parma, 43126 Parma, Italy; 17 Department of Urology, King Fahd Hospital, 23325 Jeddah, Saudi Arabia; 18 Department of Urology, Sun Yat-Sen Memorial Hospital, Sun Yat-Sen University, Guangzhou, 510120 China; 19 Department of Urology, Renmin Hospital of Wuhan University, Wuhan, 430060 China; 20 Department of Urology, Shanghai Changhai Hospital, Second Military Medical University, Shanghai, 200433 China; 21 Department of Urology, Affiliated Tumor Hospital of Guangxi Medical University, Nanning, 530022 China; 22 Department of Urology, University Hospital Southampton NHS Trust, Southampton, SO16 6YD UK; 23 Department of Urology, Hadassah Hebrew University Hospital, 91120 Jerusalem, Israel; 24 Department of Urology, Pantai Hospital, 11900 Penang, Malaysia; 25 Department of Urology, Vienna General Hospital, Medical University of Vienna, 1090 Vienna, Austria; 26 Department of Urology, Hospital de Clinicas Jose de San Martin, 1120 Buenos Aires, Argentina; 27 Department of Urology, Hamad Medical Corporation, 2001 Doha, Qatar; 28 Department of Urology, Jose R. Reyes Memorial Medical Center, 1003 Manila, Philippines; 29 Department of Nephrourology, Nagoya City University Graduate School of Medical Sciences, Nagoya, 464-0083 Japan; 30 Department of Urology, University of Turin, San Luigi Gonzaga Hospital, 10043 Orbassano, Turin, Italy; 31 Department of Urology, Klinikum Sindelfingen-Boeblingen, University of Tuebingen, 71032 Tuebingen, Germany; 32 Department of Urology, Sismanogleion General Hospital, School of Medicine, National and Kapodistrian University of Athens, 15126 Athens, Greece; 33 Department of Urology, Saint-Petersburg State University Hospital, Saint-Petersburg, Russia 194100; 34 Department of Urology and Andrology, Astana Medical University, 010000 Astana, Kazakhstan; 35 Department of Urology, Puigvert Foundation, Autonomous University of Barcelona, 08025 Barcelona, Spain; 36 Department of Urology, San Ignacio University Hospital, 110231 Bogotá, Colombia; 37 Department of Urology and Nephrology, Military Medical Academy, 1431 Sofia, Bulgaria; 38 Department of Urology, Al Zahraa Hospital University Medical Center and Lebanese University, Beirut, 10001 Lebanon; 39 Department of Urology, IRCCS San Raffaele Hospital, Ville Turro Division, 20127 Milan, Italy; 40 Department of Urology, Endourology and Stone Disease Section, University of Sao Paulo Medical School, Sao Paulo, 05508 Brazil; 41 Department of Urology, Specialty Hospital La Raza, National Medical Center of the Mexican Institute of Social Security, 97217 Mexico City, Mexico; 42 Department of Urology, UT Southwestern Medical Center, Dallas, TX 75390 USA; 43 Division of Urologic Surgery, Duke University Medical Center, Durham, NC 27705 USA; 44 Department of Urology, National University Hospital, Singapore, 119074 Singapore; 45 Department of Urology, Clinical Center of Serbia, School of Medicine, University of Belgrade, 112106 Belgrade, Serbia; 46 Department of Urology, Division of Endourology, University of Michigan, Ann Arbor, MI 48109 USA; 47 Department of Urology, Hospital de Base of the Federal District, Brasília, 70330-150 Brazil; 48 Department of Urology, Karolinska University Stockholm Sweden and Aarhus University Hospital, 17176 Stockholm, Denmark; 49 Department of Urology, Lillebaelt Hospital, University of Southern Denmark, 246000 Vejle, Denmark; 50 Department of Urology, St. Anna Hospital, 05500 Piaseczno, Poland; 51 Department of Urology, Akfa Medline Hospital, 100211 Tashkent, Uzbekistan; 52 Department of Urology, DOC University Clinic, 760000 Bishkek, Kyrgyzstan; 53 Department of Urology, Tajik State Medical University, 734003 Dushanbe, Tajikistan; 54 Department of Urology, Tte. Ettiene 215, 110309 Fernando de la Mora, Paraguay; 55 Department of Urology, Aria Apollo Hospital, Ameriat Square, 3001 Herat, Afghanistan; 56 Department of Urology, Fondazione IRCCS Ca’ Granda Ospedale Maggiore Policlinico, University of Milan, 28-20122 Milan, Italy; 57 Department of Surgery, Nepal Medical College Teaching Hospital, Jorpati, Kathmandu, 44600 Nepal; 58 Department of Urology, Istanbul Medipol University, Istanbul, 34815 Turkey; 59 Department of Surgery, Division of Urology, Western University, Schulich School of Medicine and Dentistry, London, ON N6A 5C1 Canada; 60 Department of Urology, Tongji Hospital, Tongji Medical College, Huazhong University of Science and Technology, Wuhan, 430074 China; 61 Department of Urology, Medical School, Biruni University, Istanbul, 34020 Turkey; 62 Department of Urology, University College Hospital of London, London, NW1 2BU UK

**Keywords:** Percutaneous nephrolithotomy (PCNL), Miniaturized PCNL (mPCNL), Expert consensus, Kidney stone, Operation

## Abstract

Over the past three decades, there has been increasing interest in miniaturized percutaneous nephrolithotomy (mPCNL) techniques featuring smaller tracts as they offer potential solutions to mitigate complications associated with standard PCNL (sPCNL). However, despite this growing acceptance and recognition of its benefits, unresolved controversies and acknowledged limitations continue to impede widespread adoption due to a lack of consensus on optimal perioperative management strategies and procedural tips and tricks. In response to these challenges, an international panel comprising experts from the International Alliance of Urolithiasis (IAU) took on the task of compiling an expert consensus document on mPCNL procedures aimed at providing urologists with a comprehensive clinical framework for practice. This endeavor involved conducting a systematic literature review to identify research gaps (RGs), which formed the foundation for developing a structured questionnaire survey. Subsequently, a two-round modified Delphi survey was implemented, culminating in a group meeting to generate final evidence-based comments. All 64 experts completed the second-round survey, resulting in a response rate of 100.0%. Fifty-eight key questions were raised focusing on mPCNLs within 4 main domains, including general information (13 questions), preoperative work-up (13 questions), procedural tips and tricks (19 questions), and postoperative evaluation and follow-up (13 questions). Additionally, 9 questions evaluated the experts’ experience with PCNLs. Consensus was reached on 30 questions after the second-round survey, while professional statements for the remaining 28 key questions were provided after discussion in an online panel meeting. mPCNL, characterized by a tract smaller than 18 Fr and an innovative lithotripsy technique, has firmly established itself as a viable and effective approach for managing upper urinary tract stones in both adults and pediatrics. It offers several advantages over sPCNL including reduced bleeding, fewer requirements for nephrostomy tubes, decreased pain, and shorter hospital stays. The series of detailed techniques presented here serve as a comprehensive guide for urologists, aiming to improve their procedural understanding and optimize patient outcomes.

**Supplementary Information:**

The online version contains supplementary material available at 10.1186/s40779-024-00562-3.

## Background

Global prevalence of urolithiasis has been steadily increasing [1–3]. Percutaneous nephrolithotomy (PCNL) remains the primary treatment for stones larger than 2 cm and smaller stones under specific circumstances [4]. Notably, urologists are currently expressing heightened concerns about postoperative complications, particularly hemorrhagic events associated with the conventional standard PCNL (sPCNL) procedure [5, 6].

The introduction of miniaturized PCNL (mPCNL) aimed to minimize renal parenchymal injuries and associated complications, originating in a 1993 series by Wu et al. [7], utilizing a 14–18 Fr peel-away sheath and coining the term “minimally invasive PCNL”. Subsequent contributions in the English literature include Jackman et al.’s [8] “mini-perc” technique for pediatric stones in 1998, minimally invasive PCNL (MIP) [9], microperc [10], ultra-mini-PCNL (UMP) [11], and super-mini-PCNL (SMP) [12].

Recent randomized controlled trials (RCTs) have provided substantial evidence supporting the potential superiority of mPCNL over sPCNL in treating moderate-size stones of 2–4 cm [13, 14]. Comparative analyses indicate lower postoperative pain, reduced hemoglobin drop, decreased transfusion rates, diminished nephrostomy tube utilization (including tubeless or tubeless procedures), and shorter postoperative hospital stays for mPCNLs [13–15]. However, the adoption of smaller tracts in mPCNLs has triggered debates, particularly regarding extended operation times and the potential for increased renal pelvic pressure (RPP) leading to back-flow and subsequent infection [16]. Consequently, the purported safety and efficacy superiority of mPCNL compared to conventional sPCNL remains a subject of ongoing debate. There is a lack of consensus on critical aspects of mPCNL including its definition, comparison to sPCNL, indications, preoperative work-up, intraoperative procedural nuances, and postoperative evaluation.

In this context, these factors are impeding the global integration of mPCNLs. Despite the previous publication of consensus and guidelines on PCNLs by the International Alliance of Urolithiasis (IAU) [17, 18], these documents do not specifically address mPCNLs. In this paper, IAU experts aim to present an authoritative consensus on the current landscape of mPCNL techniques in order to provide a clinical framework for practicing urologists.

## Methods

### Literature review

The study was initiated by establishing a project steering committee and assembling a team of key experts, forming an international panel from IAU. A non-systematic literature review spanning 1976 to the present was conducted by thoroughly searching PubMed, MEDLINE, Embase, and Scopus databases. This time frame was chosen to encompass the early publications on PCNL techniques [19]. Search terms included “percutaneous nephrolithotomy” “PCNL” “renal stone” “kidney stone” and “urinary tract stone”, utilizing boolean operators “AND” and “OR” for optimization. Cross-references were also carefully examined.

Preference was given to high-level studies for further evaluation, including RCTs, prospective non-randomized comparative studies, and meta-analysis. Research gaps (RGs) were identified through a systematic literature review and expert insights, which informed the development of a focused questionnaire survey specifically addressing mPCNLs while excluding commonalities with traditional PCNLs. A subsequent evaluation was conducted to finalize the consensus questionnaire (Additional file 1).

### Two-round modified Delphi survey and consensus formulation

A total of 64 experts specializing in PCNLs were identified and invited to participate in an online anonymous questionnaire survey. All participants provided informed consent and disclosed no potential conflicts of interest.

A modified Delphi method was adopted for consensus building, as utilized in a previous study [20]. The first-round survey invited participants to suggest additional items, with iterative question revisions as needed. The results of the first-round survey were compiled and sent back to participants for review in the second-round, and those completing both rounds were included in the final analysis. The Delphi process for each question concluded when agreement reached 70% or at the end of the second-round survey [21].

Following the second-round survey, an online panel meeting was convened with the project steering committee to review survey results and discuss non-consensus questions, shaping the formulation of conclusive consensus statements.

## Results

The second-round survey saw full participation from all 64 experts, resulting in a remarkable 100.0% response rate. The panel was predominantly composed of 59 males (92.2%) and 5 females (7.8%), representing diverse regions [Asia (46.9%, 30/64), Europe (32.8%, 21/64), America (15.6%, 10/64), and Africa (4.7%, 3/64)]. In terms of affiliation, the participants were associated with university teaching hospitals (70.3%, 45/64), government public hospitals (12.5%, 8/64), and private hospitals (17.2%, 11/64). Among them, 29 (45.3%) experts had experience in both mPCNL and sPCNL, 23 (35.9%) experts were solely experienced in mPCNL, and the remaining 12 (18.6%) experts had expertise only in sPCNL.

A total of 82 papers were selected for RGs extraction, and finally, 58 key questions were raised. These questions were categorized into 4 main domains: general information (13 questions), preoperative work-up (13 questions), procedural tips and tricks (19 questions), and postoperative evaluation and follow-up (13 questions). Additionally, the survey included 9 questions regarding experts’ information and PCNL experience (Additional file 1).

After the second-round survey, consensus was achieved on 30 questions as detailed in Table 1. An agreement level less than 70% was observed for 28 key questions, which focused on the preoperative evaluation, including whether mPCNL is indicated for infection stone or large burden stone, lithotripsy technique in mPCNL such as irrgation, lithotriptor, Ho:YAG laser setting and suction technique, as well as some follow-up tips. The controversy of these issues sparked heated debates and underwent thorough discussion during the online panel meeting, also summarized in the discussion section.

**Table 1:** Consensus statements and strength

Consensus statements	Strength (%)
18 Fr and 24 Fr are the recommended upper and lower cutoffs of sheath size of miniaturized PCNL (mPCNL) and standard PCNL (sPCNL), respectively	73.4
mPCNL brings less trauma over sPCNL	93.8
Less bleeding is noted in mPCNL than in sPCNL	87.5
Less pain is noted in mPCNL than in sPCNL	84.4
Nephrostomy tube is less frequently required in mPCNL than in sPCNL	85.9
Shorter hospital stay is required following mPCNL than sPCNL	84.4
The trade-off of mPCNL is a potential longer operation time when managing large stone burdens (> 4 cm)	87.5
mPCNL does not bring a higher risk of postoperative fever than sPCNL	71.9
Even though stone burden can be well weighted with stone volume, maximum stone diameter is preferred since it is the essence of convenience and easy for quality control	85.9
The stone burden is unanimously regarded as the primary criterion for deciding sheath size in PCNLs	84.4
The optimal indication for mPCNLs with < 14 Fr sheaths is 1–3 cm size stones	89.1
NCCT is the primary imaging choice before mPCNLs	92.2
General anesthesia is the most favored modality for mPCNLs, prioritizing optimal respiratory and circulatory management while minimizing patient discomfort	93.8
The prone position and supine position are the most frequently adopted positions in mPCNLs	92.2
Fluoroscopy-based guidance, either alone or combined with ultrasound, is the most recommended guidance in PCNLs	90.6
Urologists are preferred to perform the puncture rather than radiologists, provided they have received appropriate training and possess sufficient proficiency in PCNLs	93.8
One-shot dilation is the most preferred modality in mPCNLs due to its association with shorter access time and reduced radiation exposure while maintaining an equivalent complication rate	73.4
Ho:YAG laser emerges as the preferred lithotripsy in mPCNLs, either alone or in combination with pneumatic lithotripsy	76.6
Fragmentation lithotripsy technique with high-power Ho:YAG laser is preferred to low-power lasers	82.8
For stone removal in mPCNLs, the vacuum effect is the most frequently employed technique	70.3
Intraoperative serendipitously noted infection stones are not a contraindication for mPCNLs	73.4
Fluoroscopy remains the primary choice for detecting residual stones at the end of PCNLs	75.0
Tubeless PCNL is more prone to be performed in mPCNLs than in sPCNL in selected cases	70.3
Nephrostomy tube insertion depends on intraoperative findings, it can be removed within 2 d in patients following mPCNLs	79.1
A JJ stent is required at the end of PCNLs, and could be removed within 2 weeks	82.8
To assess the initial postoperative stone clearance, the recommended time for assessment is within the first postoperative week, either NCCT or KUB is available	71.9
For the conclusive stone clearance assessment, the recommended time for assessment is within postoperative 3 months, NCCT is preferred, and KUB alone is not adequate	91.5
Adequate rest and recuperation are advised after discharge, at least one week of rest is required before going back to work	76.6
Patient’s quality of life (QOL) is an important concern for both patients and urologists, regular evaluation is required, and telephone consultations are convenient and adequate for follow-up	71.9
Even though the Wisconsin stone quality of life (WISQOL) is a well-established tool for evaluating QOL in urolithiasis patients, further widespread application still requires efforts and attention from multiple parties	71.9

*PCNL* percutaneous nephrolithotomy, *NCCT* non-contrast computed tomography, *KUB* plain film of kidney, ureter, and bladder, *JJ* JJ stent, *Ho:YAG* Holmium:Yttrium Aluminum Garnet

## Discussion

PCNL has long been established as a standard procedure for managing large burden stones. However, mPCNL represents a relatively novel technique that is distinguishable from conventional sPCNL. This expert consensus marks the inaugural effort to comprehensively address and discuss the nuances of mPCNLs.

### General information

#### Definition of mPCNL

The survey addressed the diverse landscape of established mPCNL techniques (Table 2), highlighting potential complexities related to terminology. A wide range of techniques, including Chinese MIP [7], mini-perc [8], MIP [9], microperc [10], UMP [11], and SMP [12], contribute to the ongoing confusion in terminology.

**Table 2:** Current well-established mPCNL techniques

Author	Year	Term of mPCNLs	Size of the sheath (Fr)
Wu et al. [7]	1993	Chinese minimally invasive PCNL (MIP)	14–18
Jackman et al. [8]	1998	Mini-perc	13
Lahme et al. [9]	2001	MIP	15
Desai et al. [10]	2012	Microperc	4.85
Desai et al. [11]	2013	Ultra-mini-PCNL (UMP)	11–13
Zeng et al. [12]	2016	Super-mini-PCNL (SMP)	10–14

*mPCNL* miniaturized percutaneous nephrolithotomy

Given the inherently less invasive nature of both mPCNLs and sPCNL in comparison to open procedures, the term “minimally invasive PCNL” lacks precision. The survey underscored the commonality among mPCNL techniques, which involve using miniaturized instruments through smaller tracts compared to sPCNL. As a result, the term “miniaturized PCNL (mPCNL)” has emerged as a suitable descriptor, encapsulating both the minimally invasive aspect and the use of downsized equipment/sheaths in contrast to sPCNL.

Differences emerged in determining the optimal upper limit tract size for mPCNLs. In the present survey, 73.4% of participants favored an 18 Fr upper threshold, while 15.6% and 10.9% recommended 20 Fr and 22 Fr, respectively. In contrast, for sPCNL, 82.8% suggested a lower limit of 24 Fr, with only 17.2% opting for 22 Fr. The final consensus settled on using an upper cutoff of 18 Fr and a lower cutoff of 24 Fr for mPCNLs and sPCNL, respectively, aligning with established definitions [22, 23].

#### Comparison of mPCNLs to sPCNL

The comparison between mPCNLs and sPCNL has attracted increasing attention from urologists. A growing body of evidence from RCTs consistently indicates that mPCNLs demonstrate comparable safety and efficacy to sPCNL [13–15, 24–28].

A primary concern raised by 54.7% of participants in the survey was the potential drawback of prolonged operation time in mPCNLs. Meta-analyses universally suggest that mPCNLs, particularly in the treatment of staghorn calculi, require a longer operation time compared to sPCNL [24–28]. However, for moderate-sized stones (2–4 cm), the operation times of both techniques seem to be similar [13, 14, 29].

Furthermore, 93.8% of participants recognized that mPCNLs were less invasive than sPCNL, leading to reduced bleeding (87.5%), decreased postoperative pain (84.4%), and diminished need for nephrostomy tubes (85.9%). These findings are consistent with results from previous RCTs and meta-analyses emphasizing the benefits of mPCNLs, such as lower transfusion rates due to reduced renal parenchymal trauma, less frequent requirement for nephrostomy tubes, and consequently shorter hospital stays [13–15, 24–28]. The use of smaller nephrostomy tubes or tubeless procedures in mPCNLs is also correlated with decreased postoperative pain and analgesia requirements [13–15, 24–29].

Experts’ agreement in mPCNLs offers several advantages over sPCNL, including less trauma and faster recovery. However, this comes at the cost of a longer operation time, particularly when dealing with large stone burdens (> 4 cm).

### Preoperative work-up

#### Indications for mPCNL

In the early stages of mPCNL development, the primary focus was on pediatric patients [30]. Pediatric stone burdens were limited due to reduced renal caliceal system volumes [31]. Notably, initial studies such as the mini-perc series [8] and Lahme’s study [9] primarily addressed stones smaller than 2 cm in pediatric patients. As experience with mPCNLs increased, their application expanded to adult cohorts, even for larger stone burdens such as staghorn calculi [32–34].

Among the participants, 84.4% unanimously considered stone burden as the primary criterion for determining sheath size in PCNLs. Although stone burden can be effectively assessed by stone volume, 85.9% of responders preferred using maximum stone diameter due to its convenience and ease of quality control.

Furthermore, innovative techniques, such as utilizing an optical puncture needle in microperc procedures [10], have demonstrated efficacy in achieving optimal percutaneous access. Desai et al. [11] reported improved outcomes with UMP for treating stones measuring 1–2 cm. SMP with 14 Fr sheaths has been proven to be safe and effective for renal stones < 2.5 cm in pediatrics or < 3 cm in adults, particularly for lower pole stones or those not suitable for retrograde intra-renal surgery (RIRS) [12, 35]. Additionally, mPCNLs with 18 Fr suction sheaths are recommended for the treatment of stones < 5 cm [36].

In summary, mPCNLs with sheath sizes of 14–18 Fr are recommended for stones smaller than 4 cm [36–38], while other mPCNLs using sheaths smaller than 14 Fr sheaths are more suitable for stones ranging from 1 to 3 cm, particularly lower pole stones that are not suitable for shock wave lithotripsy or RIRS [8–12].

#### Preoperative assessment of stones and renal collecting system

Non-contrast computed tomography (NCCT) is essential for obtaining critical information about peri-renal organs, stone characteristics, stone location, hardness of stones, and renal parenchymal thickness. Its comprehensive insights have led to widespread acceptance of NCCT as an indispensable imaging modality prior to PCNLs [39]. A notable 92.2% of participants acknowledged CT as the primary imaging choice before mPCNLs. Additionally, 75.0% indicated that they would routinely schedule an NCCT scan.

While contrast-enhanced imaging techniques such as computed tomography urography or intravenous urography (IVU) accurately illustrate pelvic-calyceal anatomy, their dependence on sufficient split renal function limits their use in selected cases [40, 41]. Only 37.5% of participants considered contrast-enhanced imaging mandatory.

### Procedural tips and tricks

#### Anesthesia and positioning

Various anesthesia modalities, including general anesthesia, epidural anesthesia, para-vertebral block, or local anesthesia, have been employed in PCNLs [42–44]. The selection of anesthesia should take into account patient comorbidities and the anesthesiologist’s preference. General anesthesia was the predominant choice for mPCNLs (93.8%), prioritizing optimal respiratory and circulatory management while minimizing patient discomfort [42, 43, 45]. Only 1.6% of participants advocated for para-vertebral blocks.

Patient positioning for mPCNLs is influenced by individual factors and the urologist’s preference, encompassing prone, supine, lateral, or modified positions with combined antegrade and retrograde access [46, 47]. The survey revealed that the prone position (57.8%) and supine position (34.4%) were the most commonly utilized positions, while only 4.7% and 3.1% recommended lateral and modified positions, respectively. A recent meta-analysis suggests that supine PCNLs significantly reduce operation time and postoperative fever without compromising stone-free rate (SFR) [48]. However, the available puncture area in supine PCNLs is notably limited compared to the prone position [49, 50]. Modified positions, such as the prone split-leg position, have gained popularity for their ability to shorten operation time and allow simultaneous retrograde access if needed [51, 52].

#### Puncture and tract establishment

The establishment of a percutaneous tract is a crucial step in PCNL, serving two essential purposes: facilitating stone removal and minimizing the risk of severe bleeding or other tract-related complications [53]. A significant 93.8% of participants recommended that urologists perform the puncture, provided they have received appropriate training and possess sufficient proficiency in PCNLs [54].

When analyzing the guidance methods used in PCNLs, the survey findings indicated that 29.7% relied solely on X-ray, 9.4% on ultrasound alone, and 60.9% on a combination of ultrasound and X-ray. The increasing popularity of ultrasound over the past two decades can be attributed to its ability to prevent injuries to peri-renal organs, reduce radiation exposure, and provide real-time monitoring of the needle placement, thus decreasing access time [55, 56]. However, fluoroscopy is preferred for monitoring tract dilation, leading to the adoption of combined ultrasound and X-ray as an optimal approach that balances the benefits of both modalities [57].

Concerning tract dilation, various strategies have been proposed, including one-shot dilation, balloon dilation, stepwise fascial dilation, and metal telescopic dilation [58]. All these approaches have demonstrated safety and efficacy in adult patients, including those with prior open renal surgery. However, achieving a tract dilation to ≤ 18 Fr during mPCNLs is generally considered easier than reaching ≥ 24 Fr in sPCNL. One-shot dilation was the preferred method by 73.4% of participants in mPCNLs due to its association with shorter access times and reduced radiation exposure while maintaining equivalent complication rates [59].

#### Lithotripsy and intraoperative management

In terms of lithotripsy and intraoperative management, the most frequently concerned potential drawbacks in mPCNLs were prolonged operation time (54.7%), high RPP (40.6%), and the need for additional instruments (29.7%). However, none of these issues reached a consensus level of 70.0%.

Common lithotripsy techniques employed during mPCNLs include pneumatic- and laser-lithotripsy, utilizing either Ho:YAG laser or Thulium fiber laser [60–62]. Notably, the choice of lithotripsy tool does not affect SFR but does influence lithotripsy time [60, 61]. In this survey, Ho:YAG laser emerged as the preferred option (76.6%), either alone or in combination with pneumatic lithotripsy. High-power Ho:YAG laser demonstrates faster fragmentation compared to low-power lasers [63], leading to 82.8% of participants favoring this technique. Thulium fiber laser lithotripsy, recommended by only 21.9% of participants, remained unavailable in many regions.

For stone removal, the vacuum effect (70.3%) was the most commonly utilized technique. However, irrigation poses a potential challenge by increasing RPP during mPCNLs [64, 65]. Approximately 62.5% of participants observed higher RPP in mPCNLs compared to sPCNL. Importantly, the mean RPP in 14–18 Fr mPCNLs remains below 30 mmHg, which is a critical threshold for preventing pyelovenous and pyelolymphatic back-flow [66]. The use of suctioning sheaths in SMP, enhanced-SMP, and other mPCNL procedures has proven effective in decreasing RPP [36, 67, 68]. Despite concerns raised about postoperative fever in mPCNLs, it is comparable to sPCNL rates [24–28]. Based on data regarding RPP and postoperative fever [24–28, 66–68], only 28.1% of participants considered mPCNLs to have a higher risk of postoperative fever, and 26.6% viewed intraoperative serendipitously discovered infection stones as a contraindication for mPCNLs.

Recently, there has been a growing interest in the utilization of the suction technique in mPCNLs, as recommended by 40.6% of participants. This technique not only decreases RPP compared to a closed outflow system [36, 68], but also enhances lithotripsy and stone removal efficiency [36]. The advancements in laser lithotripsy and active suction techniques in mPCNLs held promise for improving SFR and treating larger stones [36, 39].

Postoperative infections are currently receiving increased attention [69–71], particularly in light of well-defined risk factors [72, 73]. Intraoperative stone culture (SC) and renal pelvic urine culture (RPUC) are considered more reliable than preoperative midstream urine culture for predicting post-PCNL fever and urosepsis, identifying pathogens, and guiding precise antibiotic therapy [74, 75]. However, the collection of SC and RPUC was not a standard practice for all the patients. Only 65.6% and 57.8% of participants would collect samples for SC and RPUC respectively, typically reserved for cases involving pyonephrosis or highly suspected infection stones. Stone fragmentation urine culture emerges as a technically feasible alternative to SC [76].

The duration of the operation is identified as a significant independent risk factor for post-PCNL complications, such as infections and bleeding [32, 77]. Although there was no consensus on the necessity of strict control over operation time in mPCNLs in this study, 43.7% of participants recommended an upper threshold of 120 min, 31.3% recommended 90 min, 9.4% recommended 60 min, and 15.6% did not propose a specific limit. The prevailing consensus suggests that operation time can be effectively managed in mPCNLs for treating medium-sized stones; however, urologists are generally advised against attempting mPCNLs for large burden stones > 5 cm.

#### Exit strategy

Prior to concluding a case, it is imperative to conduct an assessment for residual stones [78]. Retrograde or antegrade flexible nephroscopy/ureteroscopy effectively identifies residual fragments post-PCNL, while RIRS is preferred for examining a larger number of calices [79]. Endoscopic combined intra-renal surgery holds the potential to increase SFR and decrease the need for multiple tracts and associated complications [80]. Fluoroscopy remains the primary choice for detecting residual stones [81], endorsed by 75.0% of participants, while ultrasonography was favored by 21.9%, primarily due to ultrasound’s limitations in distinguishing blood clots and small residual fragments [82]. Although intraoperative CT scanning during PCNL is feasible and offers a more accurate estimation of residual stones compared to fluoroscopy [83], its widespread applicability in most hospitals is limited.

Following PCNL, various nephrostomy tubes and JJ ureteric stents have been employed to facilitate appropriate urine drainage and promote hemostasis. Due to the minimally invasive nature of mPCNLs [13–15, 24–28], it is theoretically anticipated that there will be reduced frequency or shortened durations for nephrostomy tube usage during mPCNL [13–15]. Although 93.7% of participants agreed with this viewpoint, only 70.3% expressed a preference for performing tubeless mPCNLs in selected cases. Furthermore, 46.9% continued to insert tubes in all tracts in real-world scenarios. Regarding the duration of tube placement, 79.1% of participants favored removal within 2 d. Additionally, 82.8% indicated a willingness to insert a JJ stent and remove it within 2 weeks, while 3.1% stated that they would never use a JJ stent after mPCNL. The decision to use a nephrostomy tube varied significantly across different regions and was generally dependent on intraoperative findings and urologists’ preferences. Tubeless PCNLs should be considered for selected cases without active bleeding, ureteric obstructions, or perforations of the pelvic-calyceal system [51, 84–86].

### Postoperative evaluation and follow-up

To assess the initial postoperative stone clearance, the recommended time varies, 71.9% of responders suggest within the first week after surgery. In terms of the imaging, there is no consensus; however, NCCT and KUB (plain film of kidney, ureter, and bladder) are selected as the primary options by 34.4% and 52.7% of participants, respectively. Specifically, KUB is deemed valuable for evaluating the initial stone-free status and ensuring proper drainage positioning [17, 18].

For the definitive assessment of stone clearance, 49.3% of participants recommended evaluation at 3 months postoperatively, while 42.2% suggested 1 month. The majority (59.4%) of responders preferred NCCT, with only 9.4% opting for KUB alone. Literature indicates that a 1-month timeframe may be adequate for the spontaneous passage of fragments and potential removal of JJ stents [17, 18, 87]. NCCT emerges as the most accurate modality for final stone clearance assessment, showing superior sensitivity and specificity compared to ultrasound, KUB, and IVU, particularly for radiolucent stones [88].

In our survey, there was a lack of consensus on the definition of residual fragments. Specifically, 35.9% defined stone-free as the absence of any detectable fragments, while 28.1% and 34.4% recommended cut-offs of 2 mm and 4 mm, respectively. The literature suggests a clinically insignificant residual fragment (CIRF) cut-off of 4 mm, but patients with CIRF still require close monitoring and awareness of potential progression and intervention risks [89]. Residual stones < 2 mm are demonstrated to pose a very low risk of stone-related events [90, 91].

Regular follow-up is essential for monitoring stone recurrence and assessing the patient’s quality of life (QOL). For radiopaque stones, a plain film of KUB is recommended for follow-up, while ultrasonography and IVU could be employed for radiolucent stones to minimize cumulative radiation exposure from NCCT [92]. Adequate rest and recuperation are advised after discharge [93], with 76.6% recommending at least 1 week of rest before returning to work. Telephone consultations are considered a convenient follow-up modality, supported by 76.6% of participants. Patient’s QOL is a concern for both patients and urologists, 71.9% of participants would like to assess it. Although the Wisconsin stone quality of life (WISQOL) is a well-established tool for evaluating QOL in urolithiasis patients [94], only 28.1% of participants are currently familiar with it.

It’s recognized that the number of experts contributing to this consensus is limited. Nevertheless, the participating experts are predominantly experienced in mPCNLs through their affiliation with the IAU. Moreover, even when a consensus was not reached on various aspects of mPCNLs, the emergence of diverse individual choices from this study provides valuable insights for clinical practitioners [95]. The evolving nature of mPCNLs allows for continued development and anticipates future technological refinements that may confer additional advantages. Additionally, it is acknowledged that certain choices derived from this expert consensus, based on personal experiences, may extend beyond evidence-based guidelines. This deviation is attributed to the absence of technical details in existing guidelines due to the distinct nature and protocols of various studies.

## Conclusions

mPCNL, characterized by a tract smaller than 18 Fr and an innovative lithotripsy technique, has firmly established itself as a viable and effective approach for managing upper urinary tract stones in both adults and pediatrics. It offers several advantages over sPCNL, including reduced bleeding, decreased need for a nephrostomy tube, alleviated pain, and shorter hospital stays. The detailed techniques presented here serve as a comprehensive guide for urologists to enhance procedural understanding and optimize patient outcomes.

## Supplementary Information

**Additional file 1.**

## Data Availability

The datasets used and/or analyzed during the current study are available from the corresponding author upon reasonable request.

## Abbreviations

CIRF: Clinical insignificant residual fragment
IAU: International alliance of urolithiasis
IVU: Intravenous urography
KUB: Plain film of kidney, ureter and bladder
mPCNL: Miniaturized percutaneous nephrolithotomy
MIP: Minimally invasive PCNL
NCCT: Non-contrast computed tomography
PCNL: Percutaneous nephrolithotomy
QOL: Quality of life
RG: Research gap
RCT: Randomized controlled trial
RPP: Renal pelvic pressure
RIRS: Retrograde intra-renal surgery
RPUC: Renal pelvic urine culture
SFR: Stone-free rate
SC: Stone culture
sPCNL: Standard PCNL
SMP: Super-sini-PCNL
UMP: Ultra-mini-PCNL
WISQOL: Wisconsin stone quality of life
