# Correction: International Alliance of Urolithiasis (IAU) consensus on miniaturized percutaneous nephrolithotomy

**DOI:** 10.1186/s40779-024-00579-8

**Published:** 2024-12-16

**Authors:** Guo-Hua Zeng, Wen Zhong, Giorgio Mazzon, Wei Zhu, Sven Lahme, Sanjay Khadgi, Janak Desai, Madhu Agrawal, David Schulsinger, Mantu Gupta, Emanuele Montanari, Juan Manuel Lopez Martinez, Shabir Almousawi, Vincent Emanuel F. Malonzo, Seshadri Sriprasad, Otas Durutovic, Vimoshan Arumuham, Stefania Ferretti, Wissam Kamal, Ke-Wei Xu, Fan Cheng, Xiao-Feng Gao, Ji-Wen Cheng, Bhaskar Somani, Mordechai Duvdevani, Kah Ann Git, Christian Seitz, Norberto Bernardo, Tarek Ahmed Amin Ibrahim, Albert Aquino, Takahiro Yasui, Cristian Fiori, Thomas Knoll, Athanasios Papatsoris, Nariman Gadzhiev, Ulanbek Zhanbyrbekuly, Oriol Angerri, Hugo Lopez Ramos, Iliya Saltirov, Mohamad Moussa, Guido Giusti, Fabio Vicentini, Edgar Beltran Suarez, Margaret Pearle, Glenn M. Preminger, Qing-Hui Wu, Chu Ann Chai, Khurshid Ghani, Marcus Maroccolo, Marianne Brehmer, Palle J. Osther, Marek Zawadzki, Azimdjon Tursunkulov, Monolov Nurbek Kytaibekovich, Abdusamad Abdukakhorovich Abuvohidov, Cesar Antonio Recalde Lara, Zamari Noori, Stefano Paolo Zanetti, Sunil Shrestha, Jean de la Rosette, John Denstedt, Zhang-Qun Ye, Kemal Sarica, Simon Choong

**Affiliations:** 1 Department of Urology and Guangdong Key Laboratory of Urology, First Affiliated Hospital of Guangzhou Medical University, Guangzhou, 510230 China; 2 Department of Urology, San Bassiano Hospital, 36061 Vicenza, Italy; 3 Department of Urology, Siloah St. Trudpert Hospital, 75179 Pforzheim, Germany; 4 Department of Urology, Vayodha Hospital, Kathmandu, 44600 Nepal; 5 Department of Urology, Muljibhai Patel Urological Hospital, Nadiad, 387001 India; 6 Department of Urology, Centre for Minimally-Invasive Endourology, Global Rainbow Healthcare, Agra, 282007 India; 7 Department of Urology, Stony Brook University School of Medicine, Stony Brook, NY 11794 USA; 8 Department of Urology, Icahn School of Medicine at Mount Sinai, Mount Sinai Health System, New York, NY 10029 USA; 9 Department of Urology, Fondazione IRCSS Ca’ Granda, Ospedale Maggiore Policlinico, University of Milan, 20122 Milan, Italy; 10 Department of Urology, Barcelona Clinical Hospital, 08036 Barcelona, Spain; 11 Department of Urology, Sabah Al Ahmad Urology Centre, 20005 Kuwait, Kuwait; 12 Department of Surgery, Section of Urology, Veterans Memorial Medical Center, 1110 Quezon City, Metro Manila Philippines; 13 Department of Urology, Darent Valley Hospital, Dartford, DA2 8DA UK; 14 Department of Urology, University of Belgrade, 11120 Belgrade, Serbia; 15 Department of Urology, Stone and Endourology Unit, University College London Hospitals NHS Foundation Trust, London, NW1 2BU UK; 16 Department of Urology, Hospital, University of Parma, 43126 Parma, Italy; 17 Department of Urology, King Fahd Hospital, 23325 Jeddah, Saudi Arabia; 18 Department of Urology, Sun Yat-Sen Memorial Hospital, Sun Yat-Sen University, Guangzhou, 510120 China; 19 Department of Urology, Renmin Hospital of Wuhan University, Wuhan, 430060 China; 20 Department of Urology, Shanghai Changhai Hospital, Second Military Medical University, Shanghai, 200433 China; 21 Department of Urology, the First Affiliated Hospital of Guangxi Medical University, Nanning, 530022 China; 22 Department of Urology, University Hospital Southampton NHS Trust, Southampton, SO16 6YD UK; 23 Department of Urology, Hadassah Hebrew University Hospital, 91120 Jerusalem, Israel; 24 Department of Urology, Pantai Hospital, 11900 Penang, Malaysia; 25 Department of Urology, Vienna General Hospital, Medical University of Vienna, 1090 Vienna, Austria; 26 Department of Urology, Hospital de Clinicas Jose de San Martin, 1120 Buenos Aires, Argentina; 27 Department of Urology, Hamad Medical Corporation, 2001 Doha, Qatar; 28 Department of Urology, Jose R. Reyes Memorial Medical Center, 1003 Manila, Philippines; 29 Department of Nephrourology, Nagoya City University Graduate School of Medical Sciences, Nagoya, 464-0083 Japan; 30 Department of Urology, San Luigi Gonzaga Hospital, University of Turin, 10043 Orbassano, Turin, Italy; 31 Department of Urology, Klinikum Sindelfingen-Boeblingen, University of Tuebingen, 71032 Tuebingen, Germany; 32 Department of Urology, Sismanogleion General Hospital, School of Medicine, National and Kapodistrian University of Athens, 15126 Athens, Greece; 33 Department of Urology, Saint-Petersburg State University Hospital, Saint-Petersburg, Russia 194100; 34 Department of Urology and Andrology, Astana Medical University, 010000 Astana, Kazakhstan; 35 Department of Urology, Puigvert Foundation, Autonomous University of Barcelona, 08025 Barcelona, Spain; 36 Department of Urology, San Ignacio University Hospital, 110231 Bogotá, Colombia; 37 Department of Urology and Nephrology, Military Medical Academy, 1431 Sofia, Bulgaria; 38 Department of Urology, Al Zahraa Hospital University Medical Center and Lebanese University, Beirut, 10001 Lebanon; 39 Department of Urology, IRCCS San Raffaele Hospital, Ville Turro Division, 20127 Milan, Italy; 40 Department of Urology, Endourology and Stone Disease Section, University of Sao Paulo Medical School, Sao Paulo, 05508 Brazil; 41 Department of Urology, Specialty Hospital La Raza, National Medical Center of the Mexican Institute of Social Security, 97217 Mexico City, Mexico; 42 Department of Urology, UT Southwestern Medical Center, Dallas, TX 75390 USA; 43 Division of Urologic Surgery, Duke University Medical Center, Durham, NC 27705 USA; 44 Department of Urology, National University Hospital, Singapore, 119074 Singapore; 45 Urology Unit, Department of Surgery, University Malaya Medical Center, 50603 Kuala Lumpur, Malaysia; 46 Department of Urology, Division of Endourology, University of Michigan, Ann Arbor, MI 48109 USA; 47 Department of Urology, Hospital de Base of the Federal District, Brasília, 70330-150 Brazil; 48 Department of Urology, Karolinska University Stockholm Sweden and Aarhus University Hospital, 17176 Stockholm, Denmark; 49 Department of Urology, Lillebaelt Hospital, University of Southern Denmark, 246000 Vejle, Denmark; 50 Department of Urology, St. Anna Hospital, 05500 Piaseczno, Poland; 51 Department of Urology, Akfa Medline Hospital, 100211 Tashkent, Uzbekistan; 52 Department of Urology, DOC University Clinic, 760000 Bishkek, Kyrgyzstan; 53 Department of Urology, Tajik State Medical University, 734003 Dushanbe, Tajikistan; 54 Department of Urology, Tte. Ettiene 215, 110309 Fernando de La Mora, Paraguay; 55 Department of Urology, Aria Apollo Hospital, Ameriat Square, 3001 Herat, Afghanistan; 56 Department of Urology, Fondazione IRCCS Ca’ Granda Ospedale Maggiore Policlinico, University of Milan, 28-20122 Milan, Italy; 57 Department of Surgery, Nepal Medical College Teaching Hospital, Jorpati, Kathmandu, 44600 Nepal; 58 Department of Urology, Istanbul Medipol University, Istanbul, 34815 Turkey; 59 Department of Surgery, Division of Urology, Schulich School of Medicine and Dentistry, Western University, London, ON N6A 5C1 Canada; 60 Department of Urology, Tongji Hospital, Tongji Medical College, Huazhong University of Science and Technology, Wuhan, 430074 China; 61 Department of Urology, Medical School, Biruni University, Istanbul, 34020 Turkey; 62 Department of Urology, University College Hospital of London, London, NW1 2BU UK

**Correction: Military Medical Research (2024) 11:70** 10.1186/s40779-024-00562-3

After publication of the article, it was brought to our attention that the author name Otas Durutovic was duplicated in the author list and the second one should be replaced by the author Chu Ann Chai, and the affiliations of Ji-Wen Cheng and Chu Ann Chai are incorrect, the correct author list with correct affiliations is shown below:

Guo-Hua Zeng, Wen Zhong, Giorgio Mazzon, Wei Zhu, Sven Lahme, Sanjay Khadgi, Janak Desai, Madhu Agrawal, David Schulsinger, Mantu Gupta, Emanuele Montanari, Juan Manuel Lopez Martinez, Shabir Almousawi, Vincent Emanuel F. Malonzo, Seshadri Sriprasad, Otas Durutovic, Vimoshan Arumuham, Stefania Ferretti, Wissam Kamal, Ke-Wei Xu, Fan Cheng, Xiao-Feng Gao, Ji-Wen Cheng, Bhaskar Somani, Mordechai Duvdevani, Kah Ann Git, Christian Seitz, Norberto Bernardo, Tarek Ahmed Amin Ibrahim, Albert Aquino, Takahiro Yasui, Cristian Fiori, Thomas Knoll, Athanasios Papatsoris, Nariman Gadzhiev, Ulanbek Zhanbyrbekuly, Oriol Angerri, Hugo Lopez Ramos, Iliya Saltirov, Mohamad Moussa, Guido Giusti, Fabio Vicentini, Edgar Beltran Suarez, Margaret Pearle, Glenn M. Preminger, Qing-Hui Wu, Chu Ann Chai, Khurshid Ghani, Marcus Maroccolo, Marianne Brehmer, Palle J. Osther, Marek Zawadzki, Azimdjon Tursunkulov, Monolov Nurbek Kytaibekovich, Abdusamad Abdukakhorovich Abuvohidov, Cesar Antonio Recalde Lara, Zamari Noori, Stefano Paolo Zanetti, Sunil Shrestha, Jean de la Rosette, John Denstedt, Zhang-Qun Ye, Kemal Sarica & Simon Choong.

The original publication has been updated.

